# Comparison of sexual function between laparoscopic lateral suspension and laparoscopic sacrocervicopexy with the use of the PISQ-IR questionnaire

**DOI:** 10.3389/fmed.2024.1456073

**Published:** 2024-12-06

**Authors:** Ewelina Malanowska-Jarema, Andrzej Starczewski, Mariia Melnyk, Daniel Fidalgo, Dulce Oliveira, Jean Dubuisson

**Affiliations:** ^1^Department of Gynecology, Endocrinology, and Gynecologic Oncology, Pomeranian Medical University, Szczecin, Poland; ^2^Institute of Science and Innovation in Mechanical Engineering and Industrial Engineering, Faculty of Engineering, University of Porto, Porto, Portugal; ^3^Hôpitaux Universitaires de Genève (HUG), Geneva, Switzerland

**Keywords:** pelvic organ prolapse, lateral suspension, sacrocervicopexy, sexual function, pelvic organ prolapse/incontinence sexual questionnaire—IUGA revised (PISQ-IR)

## Abstract

**Introduction and hypothesis:**

We aimed to analyze the quality of sexual life of patients with apical vaginal wall prolapse who had undergone laparoscopic lateral suspension (LLS) and laparoscopic sacrocolpopexy (LSC).

**Methods:**

We performed a secondary analysis of sexual outcomes of a previous randomized control trial comparing LLS and LSC in 89 women with symptomatic POP stage ≥ II. We evaluated sexually active (SA) and non-sexually active women (NSA) using the Pelvic Organ Prolapse/Incontinence Sexual Questionnaire-IUGA-Revised (PISQ-IR). Women were reviewed over a period of 1 year post-surgery.

**Results:**

Analysis of the entire PISQ-IR questionnaire indicates that surgical treatment of POP resulted in an improvement of the quality of sexual life in 21 (80.76%) in the group of sexually active women after LSC and in 20 (83.33%) in the group of SA patients after LLS. In both groups of patients, dyspareunia was not observed.

**Conclusion:**

In conclusion, the quality of sexual life in SA group of patients improved significantly after both surgical procedures. The quality of sexual life of surveyed women significantly improved after curing POP symptoms.

## 1 Introduction

Pelvic Organ Prolapse (POP) primarily affects women of menopausal age and has a detrimental impact on their quality of life. In addition to causing unpleasant symptoms, POP often leads to a deterioration of sexual function (1, 2).

The number of women undergoing surgery for POP increases each year (3, 4). However, studies show that sexual function differs after various surgical procedures (5–7).

Apical suspension procedures can be broadly categorized into transvaginal and abdominal approaches. Abdominal procedures can be performed via laparotomy, conventional laparoscopy, or robotic-assisted laparoscopy. Transvaginal apical suspension methods include native tissue repairs and mesh-based repairs.

### 1.1 Sacrocolpopexy

Traditionally, sacrocolpopexy has been performed through a laparotomy, known as abdominal sacrocolpopexy (ASCP). However, over the past decade, minimally invasive techniques, including laparoscopic (LSCP) and robotic sacrocolpopexy (RSCP), have become increasingly favored due to their advantages such as shorter recovery times, reduced postoperative pain, and smaller incisions compared to the open abdominal method. Laparoscopic sacrocolpopexy (LSCP) is now regarded as the gold standard for treating apical prolapse, although it requires more advanced surgical expertise. Operating near the sacral region carries potential risks, including neurological, ureteral, or vascular injury, and postoperative bowel issues are frequently reported. Moreover, periostitis, although rare, can occur due to the weak anterior longitudinal ligament at the site of sacral attachment, increasing the risk of periosteal penetration during surgery.

Compared to vaginal procedures, ASCP is associated with higher morbidity, particularly in terms of longer operative times (notably for laparoscopic or robotic approaches), extended hospital stays, delayed return to normal activities, and increased costs. Additionally, sacrocolpopexy may involve mesh-related complications, such as erosion, infection, or pain, although these complications are rare with the use of modern surgical techniques (8–15).

Laparoscopic lateral suspension (LLS) Laparoscopic lateral suspension with mesh has emerged as a promising technique, offering both excellent anatomical and functional outcomes. The uniqueness of LLS lies in the placement of the T-shaped mesh, where the lateral arms are passed through a subperitoneal tunnel along the lateral abdominal wall, exiting just above the iliac crest. This approach minimizes the risk of injury to major blood vessels, nerves, or the bowel and ensures symmetrical, tension-free suspension along the vaginal axis. LLS is primarily indicated for the treatment of anterior pelvic organ prolapse and apical descent. It is particularly useful in cases where access to the sacral promontory is challenging, such as in the presence of dense adhesions, sigmoid megacolon, or when the left common iliac vein is positioned low and partially obstructs the promontory.

Like other mesh-based procedures, LLS carries risks of mesh-related complications, including erosion, infection, and pain. Although the mesh is placed at a distance from the vaginal mucosa, these complications can still occur. While LLS has demonstrated favorable short-to medium-term outcomes, long-term data remain limited when compared to procedures such as sacrocolpopexy. Several studies and reviews have noted a higher risk of recurrence in the anterior compartment after LLS compared to sacrocolpopexy, underscoring the need for further research into its long-term efficacy (16–19).

### 1.2 Pectopexy

Pectopexy, introduced by Banerjee and Noé in 2010 (20), is a viable alternative to sacrocolpopexy for pelvic organ prolapse repair. Like sacrocolpopexy, pectopexy employs a macroporous, monofilament mesh; however, instead of attaching to the presacral ligament, the mesh is secured to the right and left pectineal ligaments, supporting the anterior and/or posterior vaginal walls. Pectopexy offers several advantages, including a shorter operative time and a lower rate of complications compared to laparoscopic sacrocolpopexy. Additionally, it is particularly beneficial for obese patients, providing a safer and more effective option than traditional sacrocolpopexy in this population. Despite its advantages, pectopexy also has certain drawbacks. One of the primary concerns is the lack of long-term data compared to sacrocolpopexy, limiting our understanding of its durability and effectiveness over time. Additionally, pectopexy may have a higher risk of anterior compartment prolapse recurrence due to its lateral mesh fixation, which may not provide as robust support for the vaginal apex as sacrocolpopexy. There is also the potential for complications such as mesh erosion, infection, and pain, which are risks associated with any mesh-based procedure. Finally, while pectopexy avoids the risks related to sacral nerve and vascular injury seen in sacrocolpopexy, it can still pose a risk to nearby structures, including the obturator nerve and vessels, during fixation to the pectineal ligaments (20–24).

### 1.3 Sacrospinous ligament fixation

Sacrospinous ligament fixation (SSLF) is a widely recognized and frequently reported transvaginal procedure for addressing apical prolapses using native tissue techniques. This minimally invasive approach offers several advantages, including reduced recovery times, making it an appealing option for women who wish to avoid abdominal surgery or who may not be suitable candidates for sacrocolpopexy. Despite its benefits, SSLF may lead to complications such as vaginal asymmetry, which may impact sexual function. Additionally, during the procedure, there is a risk of injury to critical neurovascular structures located in proximity to the surgical site. When compared to sacrocolpopexy, the recurrence of prolapse is higher (25, 26).

### 1.4 Ipsilateral uterosacral ligament fixation

Ipsilateral uterosacral ligament fixation (USLS) is a surgical technique that suspends the vaginal apex to the proximal remnants of the uterosacral ligaments via an intraperitoneal approach. This method effectively restores the vaginal axis, thereby mitigating the higher incidence of retroflexion commonly seen with sacrospinous ligament fixation (SSLF). However, patients with more severe prolapse or significant pelvic floor laxity have been noted to experience a higher recurrence rate following this procedure. Additionally, USLS carries the risk of potential injury to adjacent structures, including the ureters and nearby nerves, which necessitates careful surgical technique and consideration during the operation (27–30).

### 1.5 Transvaginal mesh procedures

Transvaginal mesh procedures involve the insertion of synthetic mesh through the vaginal wall to provide robust and durable support for prolapsed organs. This surgical approach is particularly beneficial in cases where prolapse affects not only the vaginal apex but also the anterior and/or posterior compartments. However, these procedures carry a significant risk of complications, including mesh erosion, infection, pain, and dyspareunia. The use of mesh in vaginal surgeries has also led to numerous legal challenges, prompting restrictions in some countries due to safety concerns. As a result, careful consideration of the risks and benefits is essential when evaluating the use of transvaginal mesh for pelvic organ prolapse repair (31–33).

### 1.6 McCall culdoplasty

McCall culdoplasty was not initially designed specifically for the treatment of vaginal vault prolapse, it has been shown to effectively prevent recurrence after hysterectomy. Among the various techniques for suspending the vaginal apex during vaginal hysterectomy, McCall culdoplasty is the most commonly performed procedure. This technique involves obliterating the posterior cul-de-sac and plicating the uterosacral ligaments across the midline.

A large study conducted at the Mayo Clinic demonstrated a high success rate in preventing prolapse recurrence among patients who underwent McCall culdoplasty, with the majority expressing satisfaction with their outcomes. Therefore, McCall culdoplasty appears to be an effective method for preventing vaginal vault prolapse following primary repair after hysterectomy, with minimal associated morbidity (34–37).

Obliterative surgery, including total colpocleisis and LeFort partial colpocleisis, is another option for managing apical pelvic organ prolapse (POP). However, these procedures are typically reserved for elderly women, those with significant medical comorbidities, or individuals who are no longer sexually active (38).

Several studies suggest that laparoscopic surgery for POP may confer significant benefits comparing to the vaginal approach (39–44). Unfortunately, there is a lack of consensus regarding the optimal way of surgery to preserve women’s sexual function (45).

Laparoscopic sacrocolpopexy remains the gold standard in the treatment of apical prolapse and it is recommended in sexually active patients (14, 42). Laparoscopic lateral suspension turned out to be an alternative procedure and has proved good anatomical as well as functional outcomes (46–54). However, there are only a few studies that describe the impact of laparoscopic lateral suspension on sexual function using validated questionnaires (55, 56).

Pelvic Organ Prolapse/Incontinence Sexual Questionnaire—IUGA Revised (PISQ-IR) is condition—specific measure of sexual function in women with PFD (Pelvic Floor Disorders) (57). Despite the growing demand for validated measures of sexual dysfunction, the PISQ-IR has not been widely used in patients who have undergone laparoscopic urogynaecological procedures. This underutilization highlights a significant gap in our understanding and management of sexual dysfunction in this population.

The aim of this study was to compare sexual function outcomes between laparoscopic lateral suspension and laparoscopic sacrocervicopexy, assessed before surgery and 12 months postoperatively.

## 2 Materials and methods

This retrospective observational study included 100 women referred to our department and qualified for surgery from January 2018 to December 2021. We performed a secondary analysis of sexual outcomes of a previous randomized control trial comparing LLS and LSC (53).

Preoperative data collected included age, parity, body mass index, and hormonal status.

The study inclusion criteria were: symptomatic apical prolapse stage ≥ II according to the Pelvic Organ Prolapse Quantification (POP-Q) system, sexually active women (SA), sexually not active women (NSA), all women who were able to understand and write in Polish.

The exclusion criteria included: a history of previous urogynecological surgeries, including prolapse/incontinence surgery and hysterectomy; active malignancy; posterior vaginal wall prolapse ≥ II stage.

Stress urinary incontinence was not an exclusion criterion, but patients were informed that only surgical repair of POP would be performed. 43 patients were qualified for laparoscopic sacrocervicopexy, and 46 for laparoscopic lateral suspension. All women underwent concomitant laparoscopic supracervical hysterectomy, which is a standard procedure in our department. All surgical procedures were performed by an experienced surgical team. In our study, we utilized a polypropylene mesh with a pore size of 1 mm and a product weight of 65 g/m^2^ for LLS and SCP procedures”.

The study was approved by our institutional ethic committee, and patients who met the inclusion criteria signed informed consent prior to participation in the study. Eleven patients were excluded because they did not meet all inclusion criteria or met at least one exclusion criterion.

### 2.1 The applied questionnaire

The patients completed a validated Polish questionnaire, the Pelvic Organ Prolapse/Incontinence Sexual Questionnaire—IUGA Revised (PISQ-IR) before undergoing surgery and 12 months postoperatively. The data were collected through face-to-face interviews conducted by an experienced urogynecologist (EMJ).

PISQ-IR is the disease-specific questionnaire to assess the women’s sexual function in both sexually active (SA) and inactive women (NSA) with PFD (Pelvic Floor Disorders) (57, 58).

PISQ-IR consists of two parts. Part 1, for not SA (NSA) women, and contains four domains – specific subscales (Condition – specific—NSA—CS, Partner-related—NSA—PR, Global Quality—NSA-GQ, Condition Impact—NSA-CI). Part 2, for sexually active (SA) women with six domains – specific subscales (Arousal-Orgasm—AO, Condition-specific—CS, Partner-related—PR, Desire – D, Condition Impact – CI, Global Quality—GQ).

**Figure 1:**
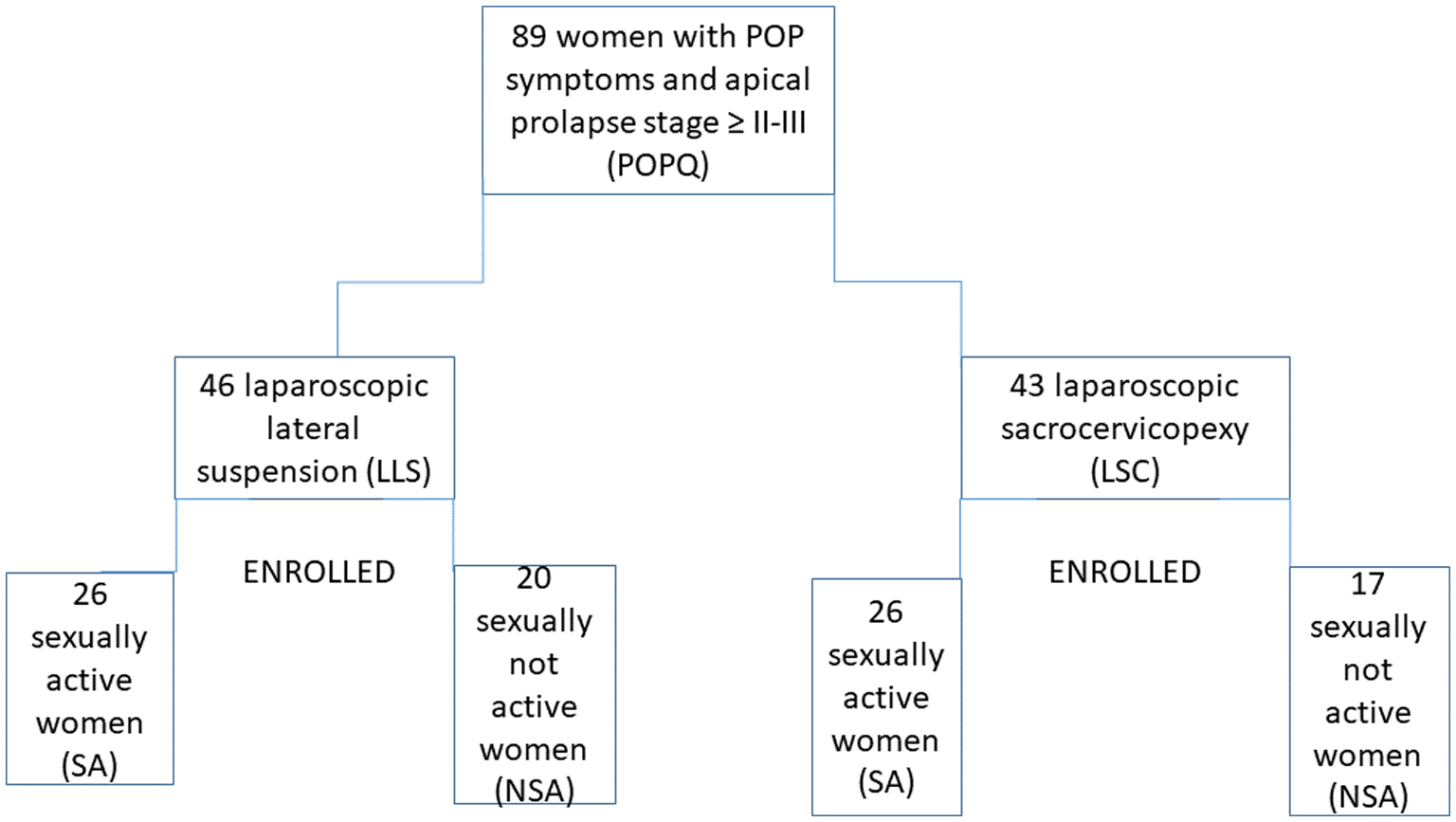
Study flowchart.

In the PISQ-IR questionnaire, the first question (Q1) describes the engagement in sexual activity and sound: “Which of the following describes you?” According to this, we enrolled patients to two groups SA and not SA. The enrolment process is shown in Figure 1.

Data was collected by face to face interview and from the patient’s medical records. Physical examination was conducted one month after surgery, including POP-Q evaluation. The information collected from patients’ medical records included anamnesis and the patients’ physical examination results. All women underwent post-operative follow-up within 3–6 months postoperatively to assess recurrent prolapse or mesh exposure or other potential complications of the surgery. A personal interview 12 months after surgery was carried out by an experienced urogynaecologist EMJ. In this interview, the patients were requested to answer a PISQ IR questionnaire.

### 2.2 Statistical analysis

Based on the collected data, a database was created using Microsoft Excel^®^ 2013 (15.0.5589.1000) MSO (15.0.5589.1000) (32-bit), from Microsoft Office Standard 2013, Microsoft Corporation, manufacture code DG7GMGF0D7FX:0002. The data were statistically analyzed using Gretl software version 2017a. Comparisons were made between LLS and LSC preoperatively and 12 months postoperatively; the *p* value was obtained using a *t*-test. The significance level was assumed to be *p* < 0.005.

## 3 Results

In the analyzed group of 89 female patients, 52 (58.42%) were sexually active (SA) and 37 (41.57%) were inactive (NSA). All of the patients were qualified for surgery because of pelvic organ prolapse (POP). We have observed significant improvement of POP after both procedures. Tables 1, 2 present anatomic outcomes before and after both surgeries.

**Table 1:** Anatomic outcomes in patients undergoing laparoscopic sacrocervicopexy with mesh.

POP-Q parameters	Preoperative		Follow-up		*p*
	Mean	SD	Mean	SD	
Aa	0.86	0.91	−1.49	1.18	0.000
Ba	1.70	1.10	−1.37	1.50	0.000
Ap	−0.72	0.73	−1.63	0.79	0.000
Bp	−0.72	0.73	−2.09	1.77	0.000
C	0.35	1.53	−5.44	2.51	0.000
gh	3.91	0.57	2.84	0.78	0.000
pb	2.33	0.81	2.63	0.62	0.022
tvl	10.00	−	10.00	−	

POP – Q – Pelvic Organ Prolapse Quantification System, SD – Standard Deviation, *p* – value <0.0001.

**Table 2:** Anatomic outcomes in patients undergoing laparoscopic lateral suspension with mesh.

POP-Q parameters	Preoperative		Follow-up		*p*
	Mean	SD	Mean	SD	
Aa	1.02	0.54	−1.65	0.95	0.000
Ba	1.91	0.51	−1.57	1.20	0.000
Ap	−0.41	1.09	−1.57	1.05	0.000
Bp	−0.46	0.98	−2.07	2.12	0.000
C	0.30	1.41	−5.39	2.75	0.000
gh	4.09	0.41	2.96	0.70	0.000
pb	2.17	0.97	2.47	0.69	0.025
tvl	10.00	–	10.00	–	

POP – Q – Pelvic Organ Prolapse Quantification System, SD – Standard Deviation, *p* – value <0.0001.

26 (60.46%) sexually active and 17 (39.53%) inactive patients were qualified for laparoscopic promontofixation surgery, while in the group of patients qualified for laparoscopic lateral suspension surgery, 26 (60.46%) were sexually active and 20 (43.47%) inactive.

In the group of 17 NSA women qualified for laparoscopic promontofixation surgery: 9 (52.94%) women had non-intercourse due to the lack of a partner (NSA-PR), 8 (47.05%) women had non-intercourse due to the lack of intercourse despite having a partner, of which in 2, the lack of desire for intercourse was the result of prolapse (NSA-CS). From the non-intercourse groups due to the surgical reduction of POP, both patients returned to sexual activity.

In a group of 26 SA women scheduled for laparoscopic promontofixation surgery, 17 (65.38%) patients stated that they “significantly” or “very much” avoid sexual activity due to the prolapse of the reproductive organ (SA-CI). The remaining 9 (34.61%) patients replied that the problem of prolapse did not determine their sexual activity. Despite this, a total of 21 (80.76%) women experienced an improvement in the quality of their sexual life after the surgery, due to the reduced feeling of discomfort associated with the improvement of anatomical conditions.

In the group of 20 NSA women qualified for lateral suspension surgery: 4 (20%) women did not have intercourse due to the lack of a partner, 16 (80%) women did not have intercourse due to the lack of willingness to have intercourse, despite having a partner, of which 9 women did not want to have intercourse caused by POP (NSA-PR, NSA-CS). From the group of women who did not have sexual intercourse due to the POP, all patients returned to sexual activity after the surgery.

In a group of 26 SA women qualified for laparoscopic lateral suspension surgery, 15 patients answered that they avoid “significantly” or “very much” sexual activity due to the POP (SA-CI). The remaining 11 patients replied that the problem of POP did not determine their sexual activity. Despite this, 20 (76.92%) women had a significant improvement in their quality of life due to the improvement of depression symptoms.

After both surgeries, no patient in the NSA and SA groups reported dyspareunia or other health problems.

Despite the lack of sexual activity due to reluctance to have intercourse or the lack of a partner, NSA women in both study groups assessed the subjective quality of sexual life (NSA-GQ-) as “sufficient and satisfactory,” both before and after surgical treatment. In response to the questionnaire question “How much does the lack of sexual activity bother you?” answered “Not at all” or “A little”.

In contrast to NSA patients who did not have sexual intercourse due to a POP, who answered the same question “It bothers me a lot” or “It bothers me a lot.” This indicates that the POP was the reason for not having intercourse.

In the group of SA patients, after both procedures, there was no significant improvement in the quality of life in terms of the feeling of orgasm or sexual desire (SA-O, SA-D domain). In the entire SA group operated on, a “positive” or “very positive” influence of the sexual partner on the perceived sexual desire was found, but the surgery did not improve the degree of sexual interest (SA-P domain).

**Figure 2:**
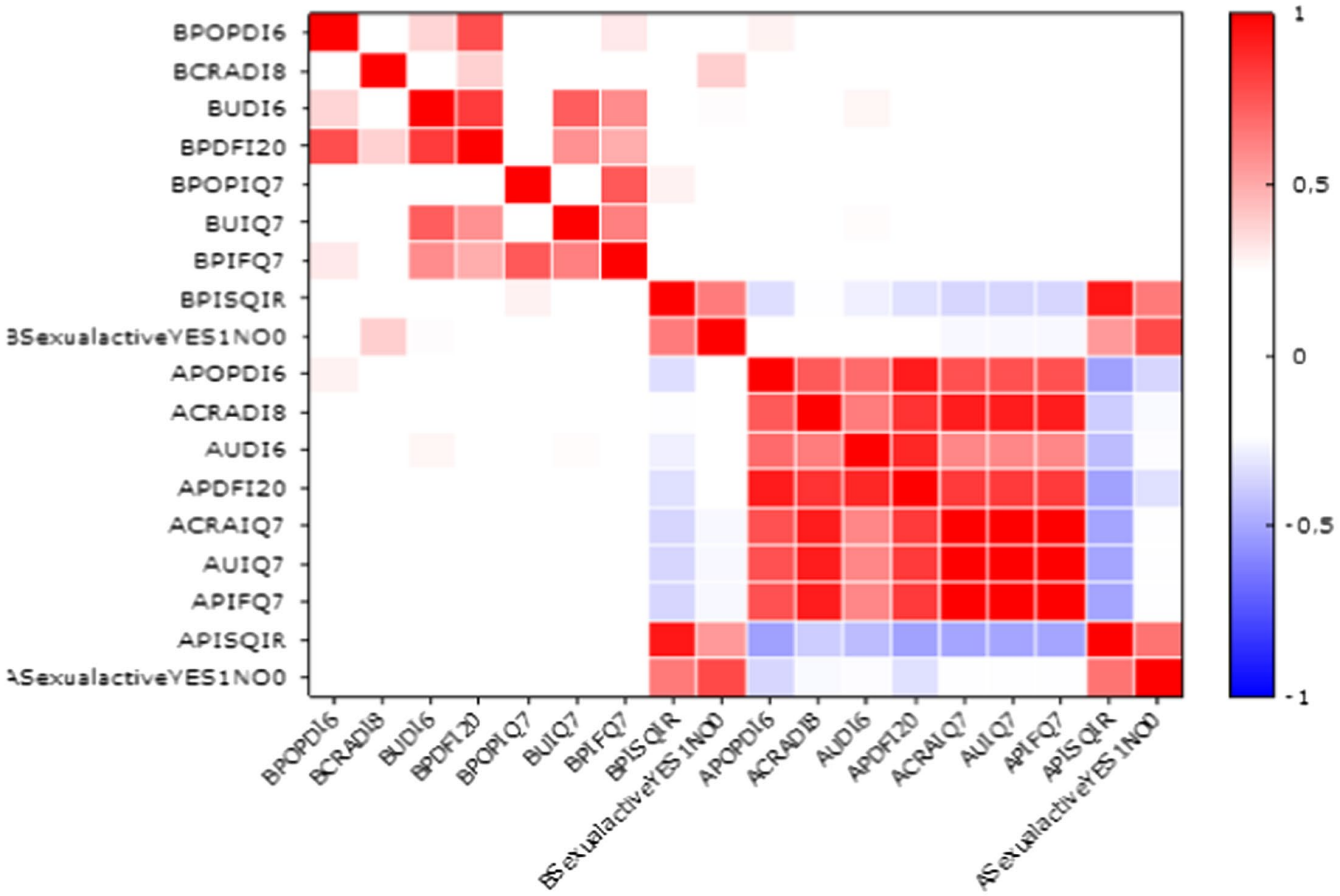
Correlation between sexual activity and symptomatic domains of questionnaire (red colors represent positive correlations, while blue colors denote negative correlations, the darker or more saturated the color—the stronger the relationship).

Analyzing the entire questionnaire, surgical treatment to correct the reduction contributed to the improvement of the quality of sexual life in 21 (80.76%) of 26 in the group of sexually active women after promontofixation surgery and in 20 (76.92%) of 26 in the group of SA patients (sexually active) after side suspension surgery (Table 3). Therefore, the quality of sexual life in this group of patients improved significantly after both surgical procedures. The treatment of the symptoms of POP significantly influenced the improvement in the quality of sexual life of the surveyed women (Figure 2).

**Table 3:** Change in sexual status at 12-months.

	Preoperatively	Postoperatively
NSA at baseline	37 (41.57%)	11(29.72%)
Change from NSA to SA	11 (29.72)	11(4%)
SA at baseline	52 (58.42%)	41 (78.84)
Change from SA to NSA	0 (0%)	0 (0%)

There was no significant statistical difference between groups in improvement in sexual quality of life after both procedures in NSA women. A total of 11 women in the NSA group returned to sexual activity.

## 4 Discussion

According to the International Continence Society (ICS) and the International Urogynecological Association (IUGA) Pelvic Organ Prolapse (POP) is defined as „the descent of one or more of the anterior vaginal wall, posterior vaginal wall, the uterus (cervix) or the apex of the vagina, or the perineum (perineal descent)” (59). The symptoms of Pelvic Organ Prolapse (POP) experienced by women can have a significant impact on their biopsychosocial, psychological, and social well-being. According to patient-reported outcome measures (PROMs), women with POP reported moderate levels of pain during sexual intercourse and low levels of bodily pain. Furthermore, POP was found to have a low to moderate impact on sleep quality, energy levels, quality of life, and sexual function domains, while its impact on physical symptoms and general health perception domains was relatively low. The results of PROMs assessing physical functioning varied widely, ranging from low to high impact (60, 61).

Sexual health, as defined by the World Health Organization, is a state of complete physical, mental, and social well-being related to sexuality. It encompasses not just the absence of disease or infirmity, but also emotional health (62). Sexual functioning is defined as “absence of difficulty moving through the stages of sexual desire, arousal, and orgasm, as well as subjective satisfaction with the frequency and outcome of individual and partnered sexual behavior” (63). Patient-reported outcome measures (PROMs) questionnaires provide a valuable patient-centered perspective on the effectiveness of surgical interventions for Pelvic Organ Prolapse (POP). However, it’s crucial to carefully choose a prolapse-specific questionnaire that is both validated and prelevant to the patient’s condition. This ensures an accurate and thorough portrayal of their experiences and outcomes.

To assess female sexual functioning, various questionnaires such as the Pelvic Organ Prolapse/Incontinence Sexual Questionnaire—IUGA Revised (PISQ-IR), Female Sexual Function Index (FSFI), International Consultation on Incontinence Questionnaire–Vaginal Symptoms Module (ICIQ-*VS*), Pelvic Organ Prolapse/Urinary Incontinence Sexual Questionnaire-12 (PISQ-12) can be used. The ICIQ-VS questionnaire may not be the best choice for obtaining a comprehensive understanding of a patient’s condition, as it solely focuses only on vaginal symptoms and is comparatively shorter (4 main questions). The Pelvic Organ Prolapse/Urinary Incontinence Sexual Questionnaire (PISQ) and its short form version, the PISQ-12, are the only current validated condition-specific female sexual function questionnaires purposively developed to assess sexual function in women with UI and/or POP (64, 65).

PISQ-12 is a shortened version of the PISQ-31 questionnaire presented in 2001. This questionnaire is used to assess sexual function in heterosexual patients with diagnosed POP and/or UI who have been sexually active over the last 6 months. It should not be used for patients who have no partner or are sexually inactive (66). The International Urogynecological Association (IUGA) Sexual Function Working Group undertook a comprehensive re-evaluation of the Pelvic Organ Prolapse/Incontinence Sexual Questionnaire (PISQ), with the primary objectives of refining its psychometric properties, expanding its applicability to women who are not sexually active and those with anal incontinence, and creating a universally applicable instrument for international use (67). These objectives have been successfully achieved with the development of the PISQ-IR, a revised and enhanced version of the original questionnaire.

PISQ-IR includes new ways of evaluating the inherent diversity of women who suffer from pelvic floor disorders (PFDs). Notably, the questionnaire incorporates gender-neutral items to evaluate the impact of a partner on sexual function. Although PISQ-IR enhances the ability to assess outcomes in women who are not sexually active and in women with anal incontinence. Importantly, this questionnaire was designed as an instrument that was directed toward international usage. The PISQ-IR questionnaire has undergone translation and validation in 11 different languages, including Polish (67).

The main focus of our study was the treatment of apical vaginal wall prolapse. There are various surgical techniques for treating apical vaginal prolapse, including open, laparoscopic, and vaginal approaches. These surgical procedures for PFD are associated with a range of side effects, some of which can be successfully avoided by selecting an appropriate method. The gold standard to treat apical prolapse is sacrocolpopexy (SCP) (10, 48). Recent research has confirmed that laparoscopic lateral suspension (LLS) is a valid and effective alternative to sacrocolpopexy (SCP) for apical pelvic organ prolapse (POP) repair. There is no significant difference in apical prolapse cure rates between LLS and SCP, indicating that LLS can achieve comparable outcomes to the SCP. The LLS seems preferable in terms of the Female Sexual Function Index, Pelvic Organ Prolapse Symptom Score, reoperation, and complications (68).

In our study those patients who were sexually active had sexual intercourse regardless of the POP. However, the improvement of anatomical conditions after surgery reduced the feeling of discomfort. Although before surgery, the majority of respondents in the group of SA women reported that POP did not affect their sexual life.

The vast majority associated the quality of sex life with a good relationship with their partner. Like other authors, we did not find any changes in the behavioral-emotional domain after surgical treatment, which assesses sexual desire and arousal, frequency of sexual activity, and the feeling of orgasm. One year after the surgery, the percentage of women with reduced sexual desire before the surgery did not change significantly after the surgery. Patients who did not have sexual intercourse before surgery mentioned age-related decreased sexual drive as the reason for this. The next cause was the POP. Surgical treatment did not improve these feelings, only the patients’ psychological comfort. It is worth emphasizing the fact that in the group of patients who did not have intercourse, some of them returned to sexual activity. This was due to the improvement in the quality of life in the range of experienced symptoms of POP and the return of the desire to have intercourse with a partner.

Numerous studies compare the postoperative results of patients who underwent LSC and anterior vaginal mesh (AVM), including impact on sexual activity or function. For example, vaginal length was greater following LSC-Cx compared to AVM. However, it is essential to note that vaginal length does not have a significant impact on female sexuality either preoperatively or postoperatively, the most important factors were “having a partner” for sexual activity and dyspareunia for sexual function (69). The persistence of dyspareunia was found to be higher after AVM (70, 71). Besides, transvaginal procedures have an increased risk of vaginal erosion, which can occur in up to 20% of patients who undergo transvaginal surgery for POP repair (72). We did not observe any cases of mesh erosion in any of the groups.

According to the available data, surgical management of POP usually results in improved or unchanged scores in sexual function, regardless of the type of procedure used.

None of the patients reported any deterioration in the quality of sexual life after both procedures. We did not observe dyspareunia after both surgeries. There were no women who became sexually inactive due to the surgery.

Our results showed that laparoscopic surgery can improve the quality of women’s sex lives by reducing symptoms associated with POP. The improvement in satisfaction with sexual life resulted from getting rid of the main problem, which was POP and the associated discomfort. Patients who had their uterine corpus removed during urogynecological surgery considered it an element that did not affect their quality of life, including sexual life. This is extremely important in the current discussion on leaving the uterine body during surgery for static disorders.

The data and results collected in this study can serve as a reference for future follow-up on the same cohort with the same tool, namely the PISQ+IR questionnaire. Including the same questionnaire in future studies containing different surgical techniques for POP repair will allow for objective and valid comparison between the operative techniques (73).

This study has several limitations. Considering the potential long-term complications associated with vaginal mesh observed in clinical practice, further investigations are warranted. Factors contributing to these complications include the inherent complexity of pelvic floor disorders, which remain inadequately understood; the biomechanical properties of the mesh, which may not be suitable for pelvic floor applications; variations in surgical techniques and the use of different modifications in operational practices across hospitals; and deficiencies in the regulatory processes for monitoring implantable medical devices. Standardization of surgical procedures is also needed.

The follow-up period is 12 months after the surgery. A longer follow-up is required to evaluate functional status for the long-term results and potential complications such as postoperative incontinence, other voiding dysfunctions, pudendal neuralgia. This study though, as mentioned, can be an initial reference point for any future follow-up. On the other hand, 12 months after surgery is an adequate period to assess POP surgeries efficacy. Our study presents important information regarding the success of laparoscopic lateral suspension pelvic organ prolapse reconstructive surgery.

## Data Availability

The raw data supporting the conclusions of this article will be made available by the authors, without undue reservation.
